# Supplementary material for: Single cell analysis of human foetal liver captures the transcriptional profile of hepatobiliary hybrid progenitors
Source: Nat Commun. 2019 Jul 26;10:3350. doi: 10.1038/s41467-019-11266-x (PMC6659636; doi:10.1038/s41467-019-11266-x)
Supplement: Supplementary file 1 — Supplementary information_new [file 41467_2019_11266_MOESM1_ESM.pdf]

Single cell analysis of human foetal liver captures the transcriptional profile of hepatobiliary hybrid progenitors

Joe M Segal[*,†,1], Deniz Kent[†,1], Daniel J Wesche[2,3], Soon Seng Ng[1], Maria Serra[1], Bénédicte Oulès[1], Gozde Kar[4], Guy Emerton[4], Samuel J I Blackford[1], Spyros Darmanis[5], Rosa Miquel[1], Tu Vinh[1], Ryo Yamamoto[2], Andrew Bonham[2], Wayel Jassem[6], Nigel Heaton[6], Alessandra Vigilante[1], Aileen King[7], Rocio Sancho[1], Sarah Teichmann[4], Stephen R. Quake[5#], Hiromitsu Nakauchi[2#], S Tamir Rashid*[#1,2].

[1]Centre for Stem Cells and Regenerative Medicine & Institute for Liver Studies, King's College London, England, WC2R 2LS, UK

[2] Institute for Stem Cell Biology and Regenerative Medicine, Stanford University School of Medicine, Stanford, CA 94305 USA

[3]Department of Microbiology and Immunology, Stanford University School of Medicine, Stanford, Palo Alto, CA 94304, USA

[4]Wellcome Trust Sanger Institute, Hinxton, UK, CB10 1SA, UK

[5]School of Engineering, Stanford University, Stanford 94350, CA, USA

[6]Institute of Liver Studies, Kings College Hospital, SE4 9RS, UK

[7]Department of Diabetes, King's College London, England, SE1 1UL, UK

†These authors contributed equally
#These authors jointly supervised this work
*Corresponding authors

Correspondence:

Joe M Segal, Email: joe.segal@kcl.ac.uk

S. Tamir Rashid, Email: tamir.rashid@kcl.ac.uk

This file includes:
Supplementary Figures 1 to 6

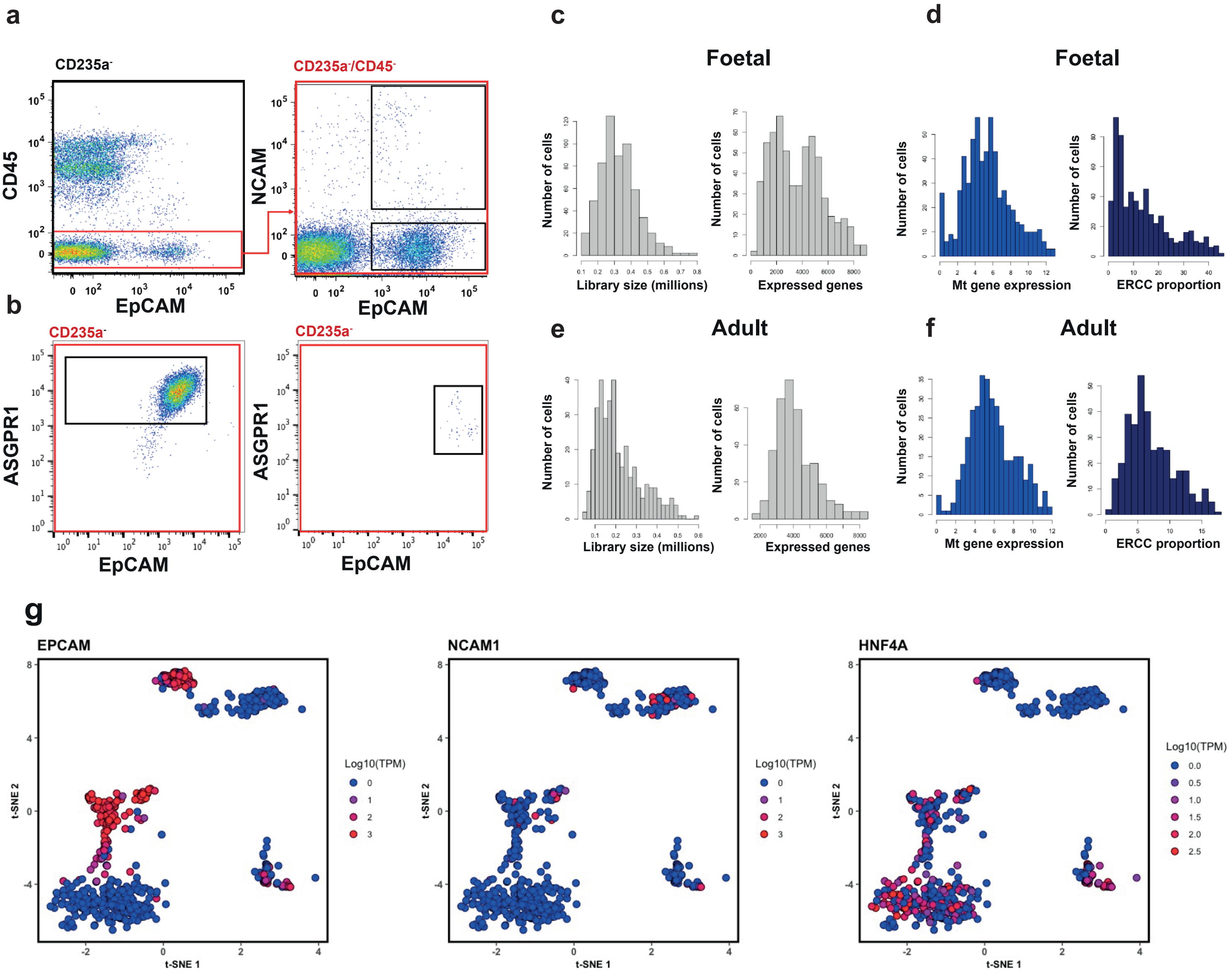
a
CD235a-
CD45
EpCAM
CD235a-/CD45-
NCAM
b
CD235a-
ASGPR1
EpCAM
c
Foetal
Number of cells
Library size (millions)
Expressed genes
d
Foetal
Mt gene expression
ERCC proportion
e
Adult
Library size (millions)
Expressed genes
f
Adult
Mt gene expression
ERCC proportion
g
EPCAM
NCAM1
HNF4A
t-SNE 1
t-SNE 2
Log10(TPM)

Supplementary Figure 1

ScRNA-seq quality control. **a** Representative FACS plots for isolation of CD235a$^-$/CD45$^-$, CD235a$^-$/CD45$^-$/EpCAM$^+$/NCAM$^-$ and CD235a$^-$/CD45$^-$/EpCAM$^+$/NCAM$^+$ cells from human foetal liver and **b** isolation of CD235a$^-$/EpCAM$^+$ and CD235a$^-$/EpCAM$^-$/ASGPR1$^+$ cells from human adult liver. Histograms of **c** library sizes and **d** number of expressed genes across all cells in the foetal and adult scRNA-seq datasets. Histograms of **e** the proportion of reads mapped to mitochondrial genes or **f** ERCC spike-in transcripts across all cells in the foetal and adult scRNA-seq datasets. **g** Transcript expression of *EpCAM, NCAM1* and *HNF4A* overlaid on the 2D t-SNE space of human liver scRNA-seq analysis. Expression is Log10(TPM). t-SNE: t-distributed stochastic neighbor embedding. TPM: Transcripts per Million. FACS: Fluorescence-activated cell sorting.

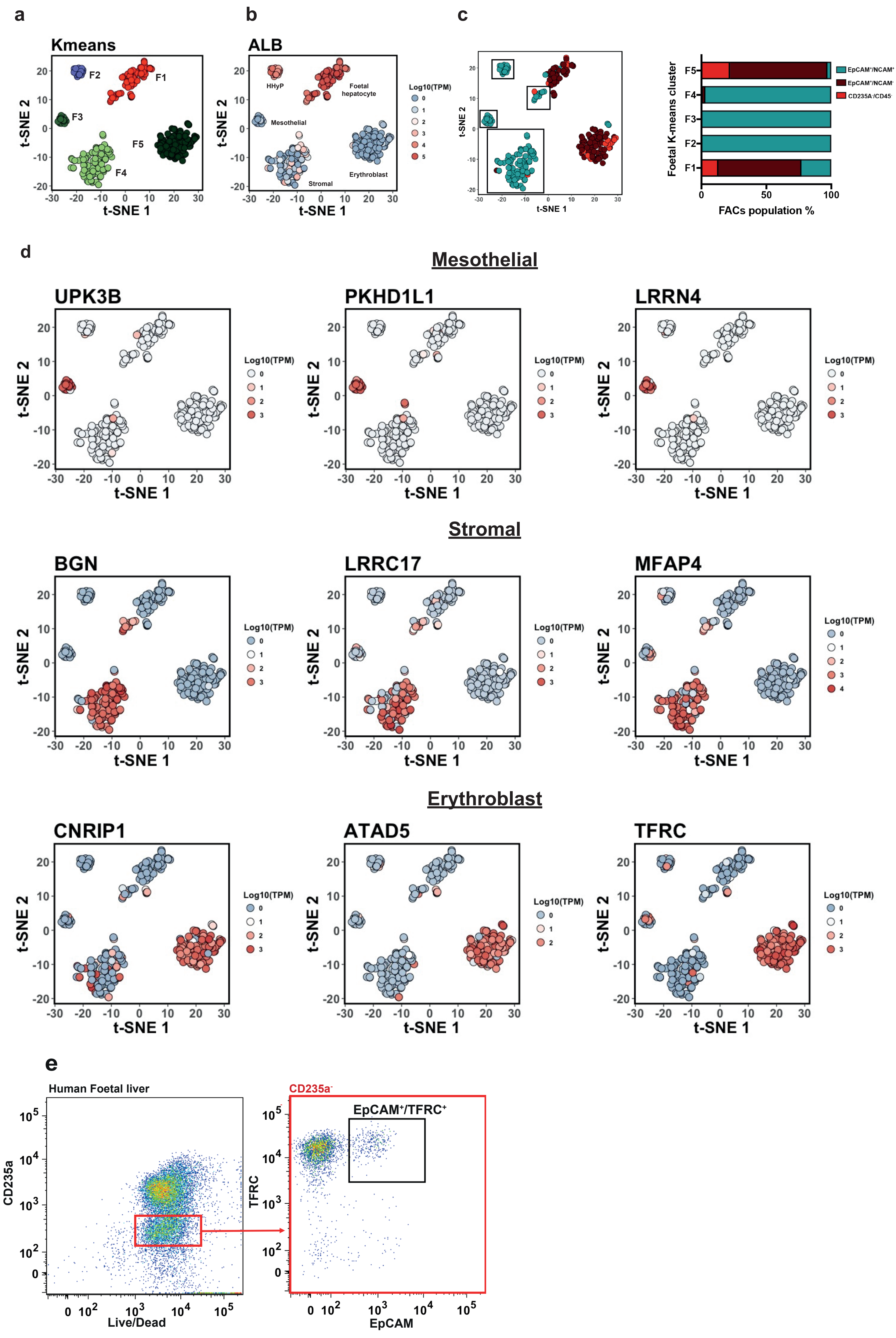

a
Kmeans
F1
F2
F3
F4
F5
t-SNE 1
t-SNE 2
b
ALB
HHyP
Foetal hepatocyte
Mesothelial
Stromal
Erythroblast
Log10(TPM)
c
Foetal K-means cluster
FACs population %
EpCAM+/NCAM+
EpCAM+/NCAM-
CD235A-/CD45-
d
Mesothelial
UPK3B
PKHD1L1
LRRN4
Stromal
BGN
LRRC17
MFAP4
Erythroblast
CNRIP1
ATAD5
TFRC
e
Human Foetal liver
CD235a
Live/Dead
CD235a-
EpCAM+/TFRC+
TFRC
EpCAM

Supplementary Figure 2:

Foetal human liver $EpCAM^+$/$NCAM^+$ cell heterogeneity. 2D t-SNE visualisation of single cells isolated from foetal human liver coloured by **a** K-means cluster, labelled as F1-F5 and **b** transcript expression of *ALB* overlaid on the 2D t-SNE space of foetal liver scRNA-seq analysis. Expression is Log10(TPM). **c** 2-D t-SNE visualisation (left panel) and bar-plots (right panel) representing the abundance (%) of each FACS populations for the foetal clusters F1-F5. **d** Transcript expression of selected markers overlaid on the 2D t-SNE space of human foetal liver scRNA-seq analysis. Expression is Log10(TPM). **e** Scatter plot of $CD235a^-$/$EpCAM^+$/$TFRC^+$ cells in human foetal liver cell FACS-analysis. Left hand plot displays Live/dead (X-axis) vs CD235a (y-axis) of all single cells. Right hand plot displays EpCAM (x-axis) and TFRC (y-axis) of live/$CD235a^-$ gated cells. t-SNE: t-distributed stochastic neighbor embedding. TPM: Transcripts per Million. FACS: Fluorescence-activated cell sorting.

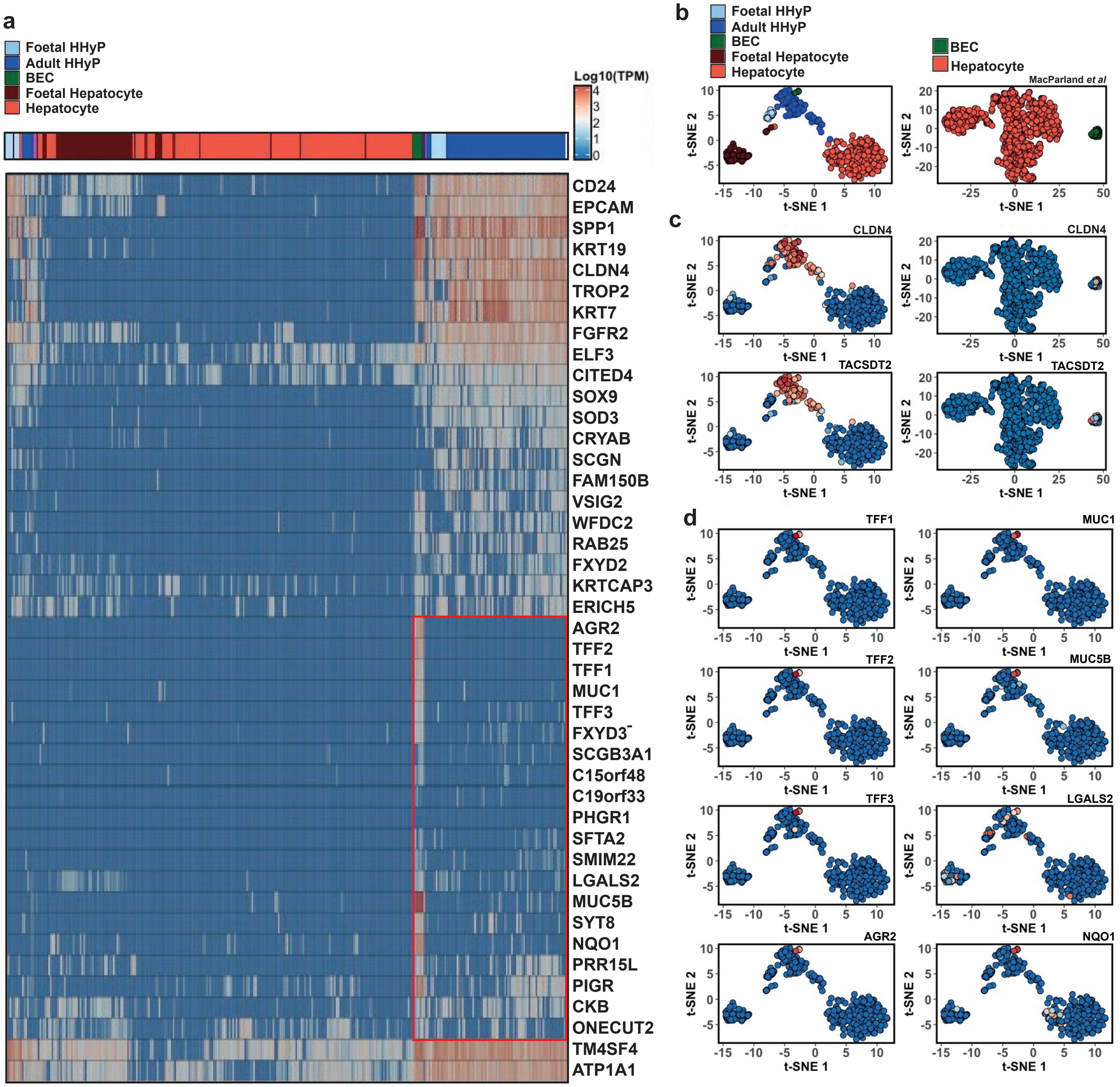
a
Foetal HHyP
Adult HHyP
BEC
Foetal Hepatocyte
Hepatocyte
Log10(TPM)
CD24
EPCAM
SPP1
KRT19
CLDN4
TROP2
KRT7
FGFR2
ELF3
CITED4
SOX9
SOD3
CRYAB
SCGN
FAM150B
VSIG2
WFDC2
RAB25
FXYD2
KRTCAP3
ERICH5
AGR2
TFF2
TFF1
MUC1
TFF3
FXYD3
SCGB3A1
C15orf48
C19orf33
PHGR1
SFTA2
SMIM22
LGALS2
MUC5B
SYT8
NQO1
PRR15L
PIGR
CKB
ONECUT2
TM4SF4
ATP1A1
b
MacParland et al
t-SNE 1
t-SNE 2
c
CLDN4
TACSDT2
d
TFF1
MUC1
TFF2
MUC5B
TFF3
LGALS2
AGR2
NQO1

Supplementary Figure 3:

Comparison of HHyP gene expression with mature human BECs. **a** Heatmap for expression of significantly upregulated genes in the mature human biliary epithelial cell (BEC) cluster from MacParland *et al.* (2018) in foetal hepatocytes, adult hepatocytes, BECs, foetal hepatobiliary hybeid progenitors (HHyPs) and adult HHyPs identified in this study [1]. **b** 2D t-SNE visualization of single cells isolated from foetal and adult human liver colored by FACS gating population (left) and MacParland *et al* Hepatocyte/BEC cell clusters (right). **c** Transcript expression of selected adult HHyP markers overlaid on the 2D t-SNE space of human foetal liver scRNA-seq analysis and MacParland *et al* (2018) hepatocyte/BEC cell clusters. Expression graded from high (red) to low (blue). **d** Transcript expression of selected mature BEC markers overlaid on the 2D t-SNE space of human foetal liver scRNA-seq analysis. Expression graded from high (red) to low (blue). Shown is gene expression patterns of publicly available sequencing data from GEO (https://www.ncbi.nlm.nih.gov/geo/). SC-RNAseq data set for comparison taken from Macparland *et al* (2018) (GSE115469)[1]. t-SNE: t-distributed stochastic neighbor embedding. FACS: Fluorescence-activated cell sorting.

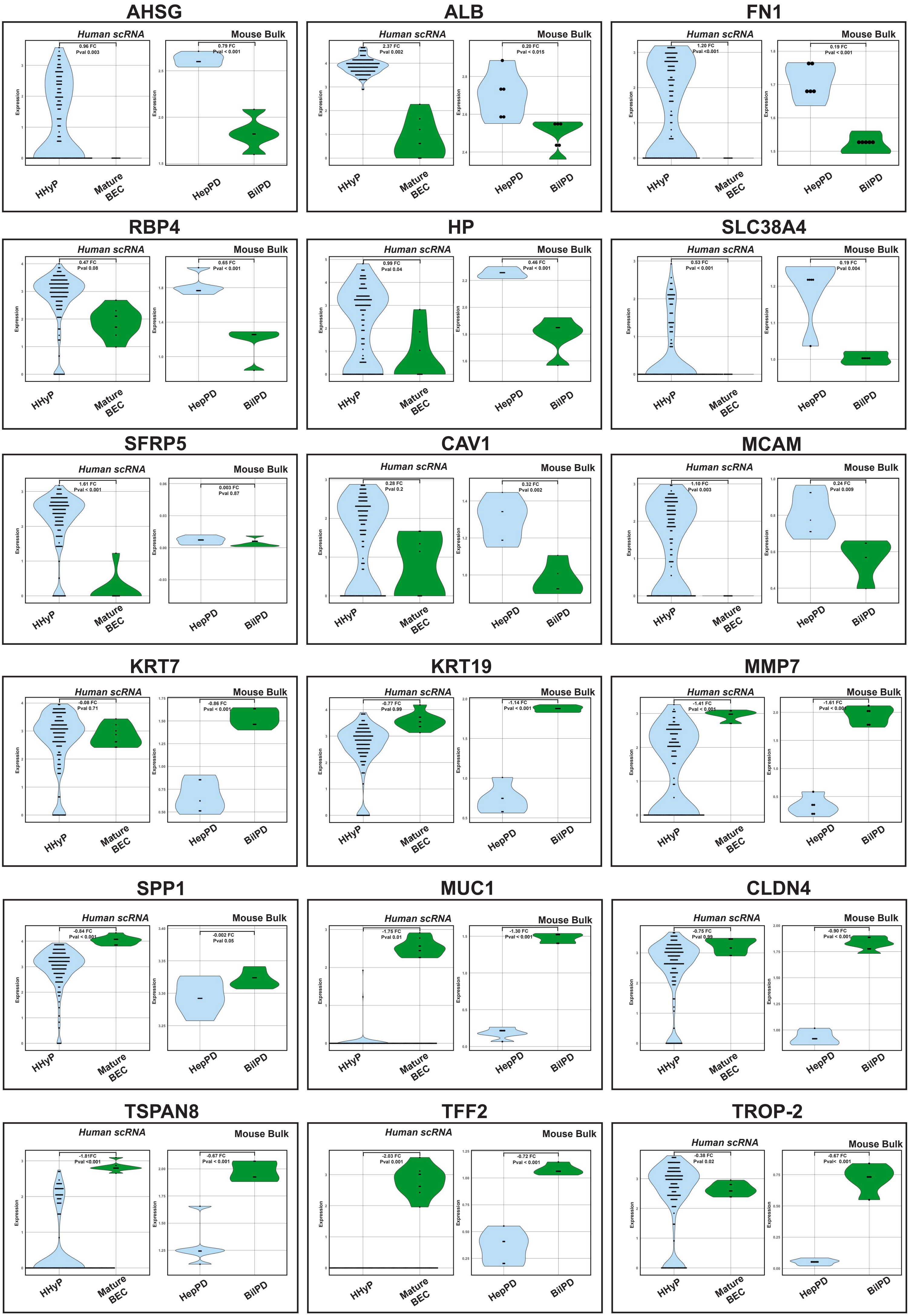
AHSG
Human scRNA
0.96 FC
Pval 0.003
Mouse Bulk
0.79 FC
Pval < 0.001
HHyP
Mature BEC
HepPD
BilPD
ALB
Human scRNA
2.37 FC
Pval 0.002
Mouse Bulk
0.20 FC
Pval < 0.015
FN1
Human scRNA
1.20 FC
Pval <0.001
Mouse Bulk
0.19 FC
Pval < 0.001
RBP4
Human scRNA
0.47 FC
Pval 0.08
Mouse Bulk
0.65 FC
Pval < 0.001
HP
Human scRNA
0.99 FC
Pval 0.04
Mouse Bulk
0.46 FC
Pval < 0.001
SLC38A4
Human scRNA
0.53 FC
Pval < 0.001
Mouse Bulk
0.19 FC
Pval 0.004
SFRP5
Human scRNA
1.61 FC
Pval < 0.001
Mouse Bulk
0.003 FC
Pval 0.87
CAV1
Human scRNA
0.28 FC
Pval 0.2
Mouse Bulk
0.32 FC
Pval 0.002
MCAM
Human scRNA
1.10 FC
Pval 0.003
Mouse Bulk
0.24 FC
Pval 0.009
KRT7
Human scRNA
-0.08 FC
Pval 0.71
Mouse Bulk
-0.86 FC
Pval < 0.001
KRT19
Human scRNA
-0.77 FC
Pval 0.99
Mouse Bulk
-1.14 FC
Pval < 0.001
MMP7
Human scRNA
-1.41 FC
Pval < 0.001
Mouse Bulk
-1.61 FC
Pval < 0.001
SPP1
Human scRNA
-0.84 FC
Pval < 0.001
Mouse Bulk
-0.002 FC
Pval 0.05
MUC1
Human scRNA
-1.75 FC
Pval 0.01
Mouse Bulk
-1.30 FC
Pval < 0.001
CLDN4
Human scRNA
-0.75 FC
Pval 0.99
Mouse Bulk
-0.90 FC
Pval < 0.001
TSPAN8
Human scRNA
-1.81FC
Pval <0.001
Mouse Bulk
-0.67 FC
Pval < 0.001
TFF2
Human scRNA
-2.03 FC
Pval 0.001
Mouse Bulk
-0.72 FC
Pval < 0.001
TROP-2
Human scRNA
-0.38 FC
Pval 0.02
Mouse Bulk
-0.67 FC
Pval< 0.001
Expression

Supplementary Figure 4:

Comparison of HHyP gene expression with mouse HepPDs . Violin plots of selected marker genes in combined foetal and adult hepatobiliary hybrid progenitors (HHyPs) and mature cholangiocyte clusters alongside Bulk RNA-seq gene expression data for mouse hepatocyte-derived proliferative ducts (HepPD) and biliary-derived proliferative ducts (BilPD) taken from[2]. Expression is Log10(TPM). For HepPD and BilPD expression n = 3 bulk RNA-seq samples. Pval calculated with student t-test). Shown is gene expression patterns of publicly available sequencing data from GEO (https://www.ncbi.nlm.nih.gov/geo/). Bulk RNA-seq data sets taken from mouse hepatocyte-derived proliferative ducts (HepPD, Tarlow *et al* 2015) and biliary-derived proliferative ducts (BilPD, Tarlow *et al* 2015) (GSE55552) [2]. TPM: Transcripts per Million.

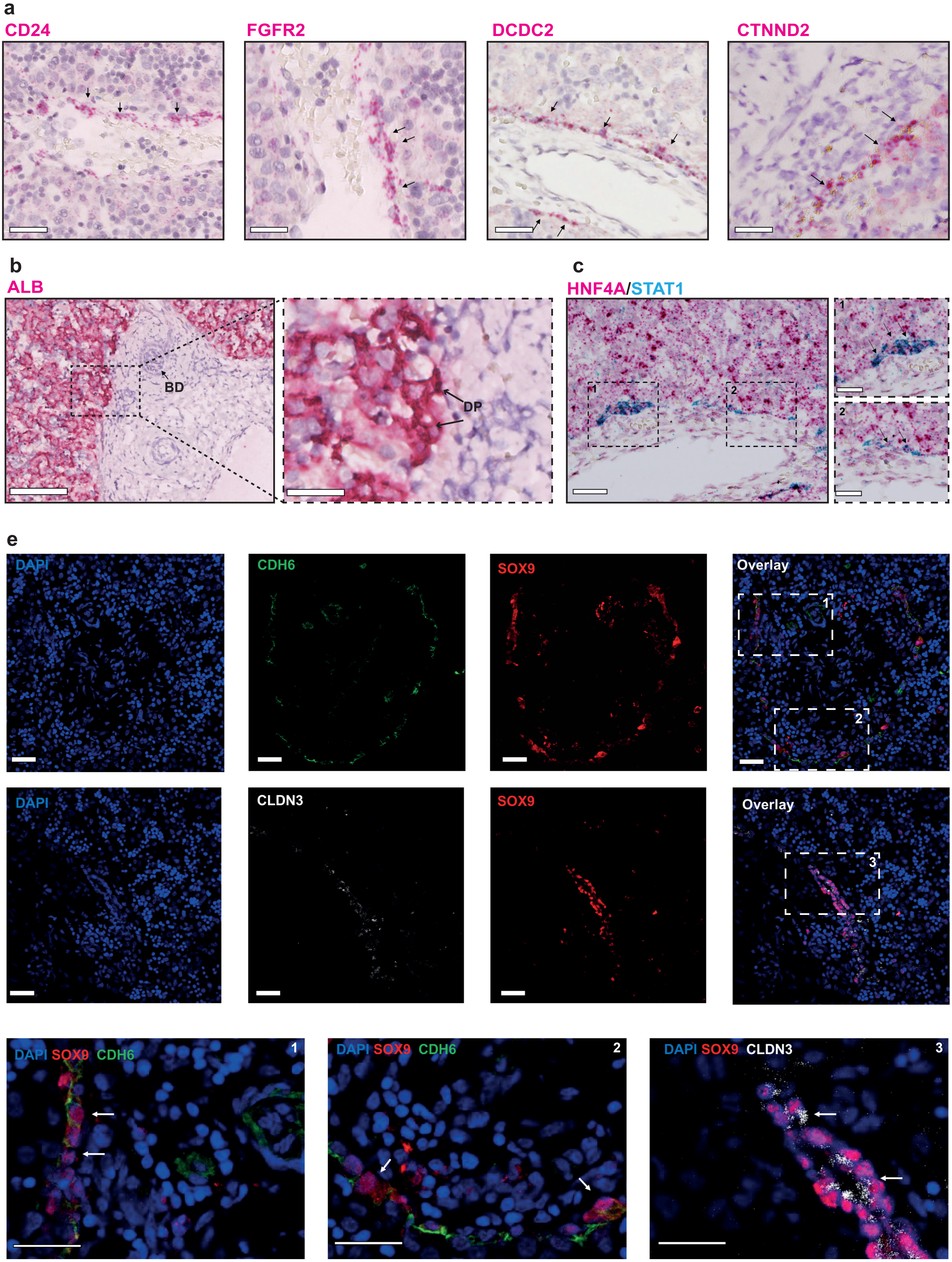
a
CD24
FGFR2
DCDC2
CTNND2
b
ALB
BD
DP
c
HNF4A/STAT1
1
2
e
DAPI
CDH6
SOX9
Overlay
DAPI
CLDN3
SOX9
Overlay
3
DAPI SOX9 CDH6
DAPI SOX9 CDH6
DAPI SOX9 CLDN3

Supplementary figure 5:

Expression of biliary and ductal plate markers in human foetal liver**. a** RNA-*ISH* for *CD24, FGFR2, DCDC2* and *CTNND2* on human 2nd trimester (15-21 pcw) foetal liver ductal plate (DP) regions. Scale bars represent 25 µm. **b** RNA-*ISH* for *ALB (red)* on human 2nd trimester (15-21 pcw) foetal liver ductal plate (DP) and bile ducts (BD) regions. Scale bars represent 50 µm. Scale bar of zoomed in region represents 25 µm. **c** Duplex RNA-*ISH* for *HNF4a* (red) with *STAT1* (green) on human 2nd trimester foetal liver DP regions (detail of red/green dots expanded in the squares). Scale bars represent 50 µm, 25 µm in blown up squares. **d** Immunofluorescence (IF) staining of CDH6 (green) and CLDN3 (white) with SOX9 (red) co-expression in 2nd trimester human foetal liver and zoomed in images of DP and BD regions. Slides counterstained in DAPI (cyan). DP and BD structures outlined white arrows . Scale bars represent 25 µm.

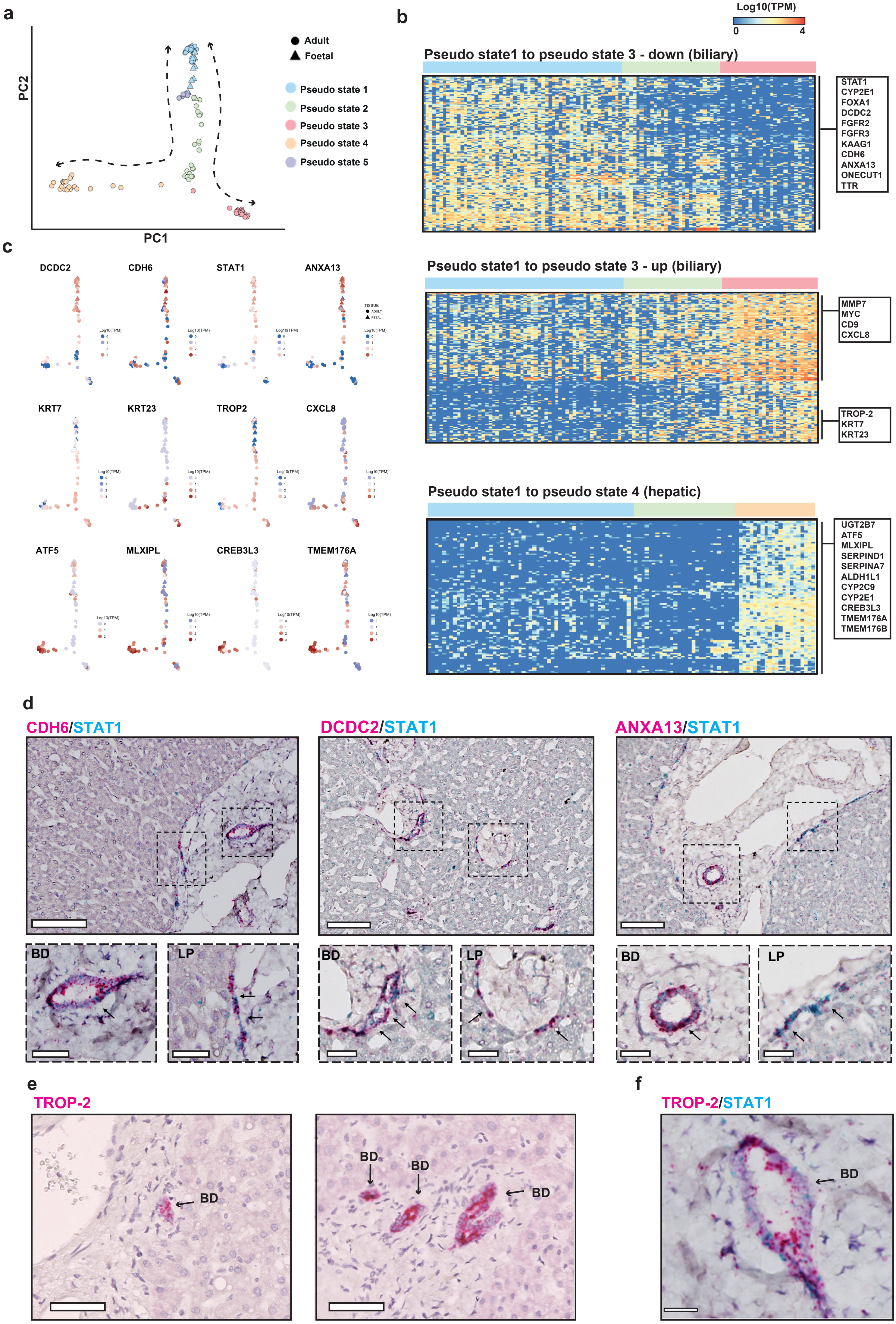
a
PC2
PC1
Adult
Foetal
Pseudo state 1
Pseudo state 2
Pseudo state 3
Pseudo state 4
Pseudo state 5
b
Log10(TPM)
0
4
Pseudo state1 to pseudo state 3 - down (biliary)
STAT1
CYP2E1
FOXA1
DCDC2
FGFR2
FGFR3
KAAG1
CDH6
ANXA13
ONECUT1
TTR
Pseudo state1 to pseudo state 3 - up (biliary)
MMP7
MYC
CD9
CXCL8
TROP-2
KRT7
KRT23
Pseudo state1 to pseudo state 4 (hepatic)
UGT2B7
ATF5
MLXIPL
SERPIND1
SERPINA7
ALDH1L1
CYP2C9
CYP2E1
CREB3L3
TMEM176A
TMEM176B
c
DCDC2
CDH6
STAT1
ANXA13
KRT7
KRT23
TROP2
CXCL8
ATF5
MLXIPL
CREB3L3
TMEM176A
d
CDH6/STAT1
DCDC2/STAT1
ANXA13/STAT1
BD
LP
e
TROP-2
BD
f
TROP-2/STAT1
BD

Supplementary figure 6:

Pseudo-lineage analysis of foetal and adult HHyPs. **a** PCA plot of Monocle pseudo-time analysis on all cells of foetal and adult hybrid hepatobiliary progenitor (HHyP) cluster, coloured by pseudo state. **b** Heat maps of significantly expressed genes between pseudo states 1,2,3 (biliary lineage) and 1,2,4 (hepatic lineage). Gene expression in Log10(TPM). **c** PCA plots of Monocle pseudo-time analysis on all cells of foetal and adult HHyPs with overlay of selected gene transcript expression. Gene expression in Log10(TPM). **d** Duplex RNA-*ISH* for *CDH6*, *DCDC2* and *ANXA13* (red) with *STAT1* (blue) in adult human liver bile duct (BD) and limiting plate (LP) structures. Detail of the red dots expanded in the squares. Scale bars represent 50 µm. Scale bars of zoomed in region represent 25 µm. Single-plex RNA-*ISH* for **e** *TROP-2* in human adult liver. Scale bars represent 50 µm (left) and 25 µm (right). **f** duplex RNA-ISH for *TROP-2* (red) with *STAT1* in adult human liver bile duct (BD) structures. Scale bar represents 50 µm. PCA: Principal components analysis. TPM: Transcripts per million.

References

1 MacParland, S. A. *et al.* Single cell RNA sequencing of human liver reveals distinct intrahepatic macrophage populations. *Nat Commun* **9**, 4383, doi:10.1038/s41467-018-06318-7 (2018).

2 Tarlow, B. D. *et al.* Bipotential adult liver progenitors are derived from chronically injured mature hepatocytes. *Cell Stem Cell* **15**, 605-618, doi:10.1016/j.stem.2014.09.008 (2014).
