# Supplementary material for: Single cell analysis of human foetal liver captures the transcriptional profile of hepatobiliary hybrid progenitors
Source: Nat Commun. 2019 Jul 26;10:3350. doi: 10.1038/s41467-019-11266-x (PMC6659636; doi:10.1038/s41467-019-11266-x)
Supplement: Supplementary file 3 — Description of Additional Supplementary Files_new [file 41467_2019_11266_MOESM3_ESM.docx]

**Description of Additional Supplementary Files**

File Name: **Supplementary Data 1. Adult hepatocyte significantly up regulated genes**

Description:

Significantly up regulated genes in adult hepatocytes identified in single cell RNA-seq analysis compared to all other cells. Fold change cut of >1.5 and p-value of <0.05 using student t-test.

File Name: **Supplementary Data 2. Adult hepatobiliary hybrid progenitors (HHyP) significantly up regulated genes**

Description:

Significantly up regulated genes in adult hybrid hepatobiliary progenitors (HHyPs) identified in single cell RNA-seq analysis compared to all other cells. Fold change cut of >1.5 and p-value of <0.05 using

student t-test.

File Name: **Supplementary Data 3. Foetal hepatocyte significantly up regulated genes**

Description:

Significantly up regulated genes in foetal hepatocytes identified in single cell RNA-seq analysis compared to all other cells. Fold change cut of >1.5 and p-value of <0.05 using student t-test.

File Name: **Supplementary Data 4. Foetal hepatobiliary hybrid progenitor (HHyP) significantly up regulated genes**

Description:

Significantly up regulated genes in foetal hepatobiliary hybrid progenitors (HHyPs) identified in single cell RNA-seq analysis compared to all other cells. Fold change cut of >1.5 and p-value of <0.05 using

student t-test.

File Name: **Supplementary Data 5. Adult biliary epithelial cells (BECs) significantly up regulated genes**
Description:

Significantly up regulated genes in adult biliary epithelial cells (BECs) identified in single cell RNA-seq analysis compared to all other cells. Fold change cut of >1.5 and p-value of <0.05 using student t-test.
